# Modulating Subcellular Localization to Preserve the Stability and Functionality of Intracellular Nanobodies

**DOI:** 10.3390/antib14040088

**Published:** 2025-10-16

**Authors:** Wenli Sun, Keke Huang, Yaping Cheng, Ailing Huang, Yu Kong, Jun Lu, Tianlei Ying, Yanling Wu

**Affiliations:** 1 Key Laboratory of Medical Molecular Virology (MOE/NHC/CAMS), Shanghai Institute of Infectious Disease and Biosecurity, School of Basic Medical Sciences, Fudan University, Shanghai 200032, China; 22111010039@m.fudan.edu.cn (W.S.);; 2 Shanghai Engineering Research Center for Synthetic Immunology, Fudan University, Shanghai 200032, China; 3 Auckland Bioengineering Institute, University of Auckland, Auckland 1010, New Zealand; 4 Department of Food and Agriculture Technology, Yangtze Delta Region Institute of Tsinghua University, Jiaxing 314006, China; 5 Shanghai Key Laboratory of Lung Inflammation and Injury, Zhongshan Hospital, Shanghai 200032, China

**Keywords:** nanobody, subcellular localization, localization motif, protein stability, intracellular delivery

## Abstract

**Background:** Antibodies have revolutionized therapeutics and diagnostics, but their applications are largely restricted to extracellular targets due to challenges in intracellular delivery and stability. Nanobodies, with their small size and lack of disulfide bonds, hold great promise for intracellular use but face challenges such as aggregation and rapid degradation in the cytosol. **Methods:** To overcome this, we engineered nanobodies by fusing them with subcellular localization motifs to redirect their localization within cells, including the mitochondrial surface, endoplasmic reticulum surface, endomembrane system, and cytoskeleton. **Results:** Our results demonstrate that nanobodies located in the cytoskeleton or endomembrane exhibit significantly reduced degradation rates and enhanced stability, while maintaining their target-binding capacity. Mechanistically, these modifications lowered ubiquitination levels and prolonged functional activity. **Conclusions:** This work provides a novel strategy to enhance the intracellular stability and efficacy of nanobodies, expanding their potential applications in functional proteomics, disease research, and therapeutic development.

## 1. Introduction

Recently, intracellular antibodies have emerged as valuable tools for probing the functions of intracellular proteins. They have been successfully applied to modulate protein–protein interactions involving intracellular targets associated with cancer and neurodegenerative diseases [1]. Compared with gene knockdown approaches such as antisense oligonucleotides (ASOs), CRISPR, or siRNA, intracellular antibodies can directly modulate protein function at the protein level, including key pathological proteins such as α-synuclein, tau, huntingtin, and TDP-43 [2,3]. Moreover, intracellular antibodies offer unique advantages when targeting post-translational modifications, specific functional domains, or conformational states of intracellular proteins, especially those with flexible structures and lacking druggable pockets [4,5]. However, the reducing environment of the cytosol poses a challenge for proper folding and assembly of full-length antibodies or antibody fragments [6,7].

Nanobodies, derived from the variable domain of heavy-chain-only antibodies (HCAbs) naturally found in camelids, represent the smallest functional antigen-binding fragments of antibodies [8]. Compared to conventional monoclonal antibodies, nanobodies possess only ~1/10 of the molecular weight. In addition, they exhibit several advantageous properties, including high epitope specificity, ease of production, exceptional stability under extreme conditions, and superior tissue penetration. These characteristics have facilitated the widespread application of nanobodies in both medical diagnostics and therapeutic interventions, particularly in cancer, autoimmune diseases, infectious diseases, and neurodegenerative disorders [9,10]. To date, the U.S. Food and Drug Administration (FDA) has approved two nanobody-based drugs: Caplacizumab and Ciltacabtagene autoleucel [11,12]. More importantly, nanobodies have emerged as the primary format for intracellular applications, due to their simple structure and lack of disulfide bonds [2,3,13]. Nonetheless, many nanobodies are intrinsically unstable in the intracellular cytoplasm [14,15]. Direct methods for selecting nanobodies with high intracellular stability are lacking, and the identified candidates still require evaluation or further engineering [15,16,17].

Protein homeostasis in the intracellular environment is maintained by a highly sophisticated network comprising around 2000 proteins, which coordinate protein synthesis, folding, conformational maintenance, trafficking and degradation [18,19]. Protein folding and trafficking are tightly regulated processes central to the maintenance of functional intracellular protein levels [18]. Notably, around two-thirds of proteins must be transported from their site of synthesis in the cytosol to their site of function within specific subcellular compartments [20]. Mislocalized or misfolded proteins can accumulate in the cytosol, exert toxic effects, and are rapidly eliminated by proteolytic systems [21,22]. Furthermore, protein half-life has been reported to vary across subcellular compartments: mitochondrial proteins tend to exhibit longer lifespans than cytosolic ones, while structural components such as nuclear scaffolds and cytoskeletal proteins are typically even more long-lived [23,24,25,26]. These observations suggest that altering the subcellular localization of exogenous proteins expressed in the cytoplasm, without affecting their function, may influence their stability and fate within the cell.

Here, we propose a broadly applicable strategy to enhance the stability of nanobodies in the cytoplasm by fusing them with localization motifs that direct them to different subcellular compartments, while maintaining their orientation toward the cytoplasmic environment. Initially, we employed a GPS-based system to evaluate nanobody stability within cells [27,28,29]. Nanobodies targeted to the cytoskeleton or endomembrane system exhibited significantly enhanced intracellular accumulation and stability. These findings were further substantiated at the molecular level by measuring nanobody degradation rates and levels of ubiquitination. We then functionally validated that nanobodies targeting intracellular antigens retained their activity after relocalization, with prolonged intracellular efficacy. Collectively, these findings provide a foundation for a novel and generalizable approach to enhance the intracellular performance of nanobodies, facilitating their broader application in both basic research and therapeutic development.

## 2. Results

### 2.1. Localization Motifs Mediate Subcellular Localization of Fusion Protein

Previous studies have demonstrated substantial differences in protein half-life across subcellular compartments [23,24,25,26]. Therefore, we investigated whether anchoring exogenous proteins to intracellular structures alters their degradation rates compared to their freely cytosolic counterparts. Furthermore, to preserve protein functionality, localization motifs were selected to anchor proteins specifically to the cytosolic-facing side of intracellular structures.

**Figure 1:**
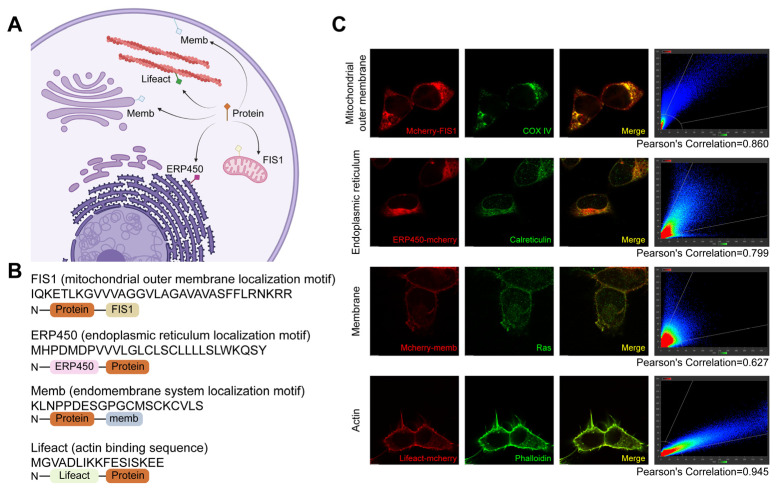
Subcellular localization of proteins directed by localization motifs fusion. (**A**) Schematic illustration demonstrating localization of exogenous proteins to specific subcellular structures through fusion with localization motifs. Arrows indicate the final localization sites, including the mitochondrial outer membrane, endoplasmic reticulum (ER) surface, endomembrane system surface, and cytoskeleton. Created with BioRender (https://www.biorender.com/, accessed on 4 September 2025). (**B**) Localization motifs and expression vector design. The FIS1 is derived from the C-terminal transmembrane segment of yeast Fis1 protein; ERP450 consists of a 27-amino-acid ER-targeting motif from cytochrome P450; Memb is derived from the C-terminal region of HRAS; Lifeact is a 17-amino acid peptide from the N-terminus of the actin-binding protein ABP140. (**C**) Co-localization analysis of mCherry fused with individual localization motifs and subcellular structures. Strong co-localization was observed, with Pearson’s correlation coefficients > 0.6. Scale bar, 5 μm.

Here, we selected four representative subcellular structures for targeted anchoring, including the mitochondrial outer membrane, endoplasmic reticulum (ER) surface, endomembrane system surface, and cytoskeleton (Figure 1A). The localization was primarily achieved by fusing endogenous localization motifs derived from endogenous proteins at these sites, with the exception of the cytoskeleton, where the actin-binding peptide Lifeact was used to avoid perturbing native cytoskeletal dynamics (Figure 1B). These localization motifs were fused to the mCherry-labeled protein to redirect their distribution from the nucleocytoplasm to specific subcellular compartments. Immunofluorescence colocalization results demonstrated successfully redirected mCherry to the mitochondrial surface (FIS1) [30,31], ER surface (CYP450) [32], endomembrane system (memb) [33,34], and cytoskeleton (Lifeact) [35] (Figure 1C).

### 2.2. Localization Motifs for the Endomembrane System or Cytoskeleton Improves Intracellular Accumulation of Exogenous Proteins

**Figure 2:**
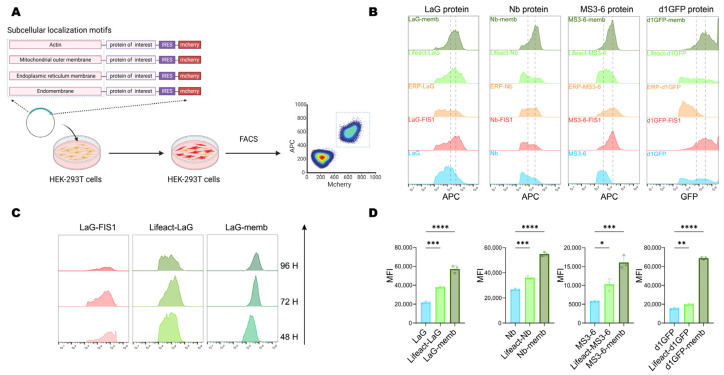
Protein level and accumulation induced by subcellular localization motifs. (**A**) Schematic diagram of the workflow for analyzing the accumulation efficiency of exogenous proteins in cells. Plasmids carrying target proteins with different subcellular localization signals were transiently transfected into HEK-293T cells, and the content of the target protein in double-positive cells was analyzed by flow cytometry after 72 h. Created with BioRender (https://www.biorender.com/, accessed on 4 September 2025). (**B**) Flow cytometry analysis of the accumulation efficiency of exogenous proteins fused with different localization motifs. The unfused construct served as a control. The two dashed lines represent the mean fluorescence intensity (MFI) of the unfused construct and memb fusion, respectively. (**C**) Prolonged cell culture post-transfection to observe the content of target proteins localized to the mitochondrial outer membrane, intracellular membranes, and cytoskeleton. (**D**) Flow cytometry analysis of the mean fluorescence intensity (MFI) for target proteins localized to endomembrane and cytoskeleton in HEK-293T cells 72 h after transfection. Mean values ± SD of three independent biological experiments. Statistical analyses of LaG proteins (**** *p* < 0.0001 and *** *p* = 0.0003), Nb proteins (**** *p* < 0.0001 and *** *p* = 0.0001), MS3-6 proteins (*** *p* = 0.0002 and * *p* = 0.0127), and d1GFP proteins (**** *p* < 0.0001 and ** *p* = 0.0032) were carried out with one-way ANOVA followed by Tukey’s multiple comparisons test.

Next, we further investigated the effect of relocalization on the protein level. The validated localization motifs were engineered at either the N- or C-terminus of the exogenous proteins. To enable comparative analysis, an internal ribosome entry site (IRES) was used to drive cap-independent expression of a downstream mCherry reporter, allowing simultaneous detection of transfected cells and normalization of transfection efficiency. Cells were transiently transfected with constructs carrying distinct localization motifs, and at indicated timepoints, samples were collected and analyzed by flow cytometry to compare fluorescence intensities of double-positive cells, thereby assessing the influence of localization motifs on exogenous protein stability (Figure 2A).

Given concerns that long endogenous half-lives might obscure observations, we initially utilized a short-lived GFP mutant (d1GFP, half-life ~1 h) [27]. Results revealed significant protein accumulation mediated by the membrane-targeting (memb), actin-binding (Lifeact), and mitochondrial outer membrane-targeting (FIS1) motifs, compared to the untagged cytosolic form. Given the importance of antibody stability for intracellular therapeutic applications, we next evaluated the generalizability of this strategy using three antibody formats: LaG (a GFP-targeting dimeric nanobody) [36], MS3-6 (a STAT3-targeting nanobody mimic) [37], and Nb (targeting viral-RNA-dependent RNA polymerase) [38]. Notably, all three showed enhanced intracellular accumulation when fused with memb, Lifeact, and FIS1 (Figure 2B).

To further compare the effects of different localization motifs, we monitored protein levels at 48-, 72-, and 96-h post-transfection. Temporal analysis revealed that the mitochondria-targeted protein underwent progressive degradation, whereas endomembrane (memb) and cytoskeleton-localized (Lifeact) proteins remained stable expression over time (Figure 2C). Flow cytometry-based quantitative analysis of the mean fluorescence intensity of the four target proteins further confirmed that these two subcellular localizations significantly promoted protein accumulation, increasing the levels of their respective untagged counterparts by 2- to 3-fold (Figure 2D). Together, these results demonstrated that endomembrane (Memb) and cytoskeleton (Lifeact) targeting peptides exhibited the most pronounced enhancement of intracellular protein accumulation, and served as potent regulators of intracellular protein stability.

### 2.3. Membrane- and Cytoskeleton- Targeting Boost Cytoplasmic Accumulation of Exogenous Proteins via Enhanced Stability

**Figure 3:**
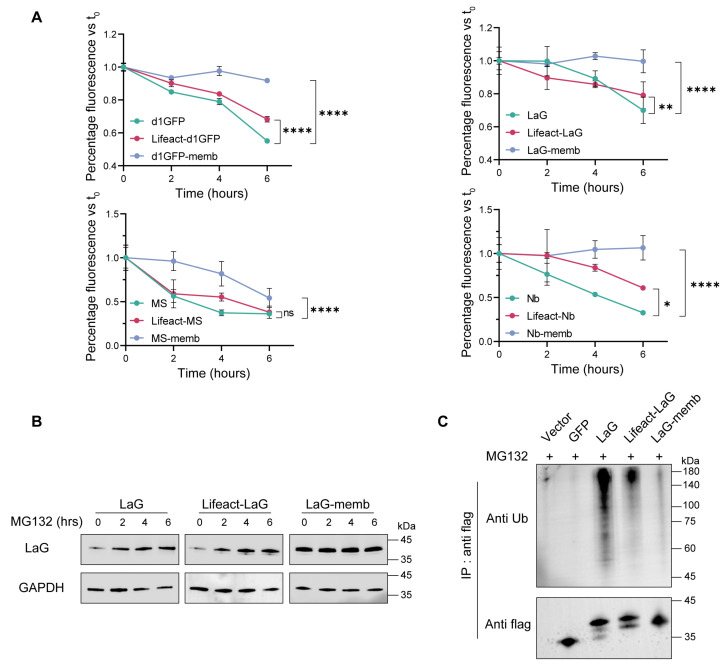
Subcellular targeting to the endomembrane or cytoskeleton reduces the degradation rate and ubiquitination level of exogenous proteins. (**A**) Protein degradation rates under different localization motifs. HEK293T cells were transiently transfected with plasmids encoding various subcellular localization motifs and subsequently treated with the protein synthesis inhibitor cycloheximide (CHX). Samples were collected at 0-, 2-, 4-, or 6-h post-treatment, and the mean fluorescence intensity of the target protein in cells was measured. The fluorescence intensity at 0 h was normalized to 100% for each construct. Mean values ± SD of three independent biological experiments. Statistical analyses of d1GFP proteins (**** *p* < 0.0001), LaG proteins (**** *p* < 0.0001 and ** *p* = 0.0010), MS3-6 proteins (**** *p* < 0.0001 and ns = 0.1216), and Nb proteins (**** *p* < 0.0001 and * *p* = 0.0151) were carried out with two-way ANOVA followed by Tukey’s multiple comparisons test. (**B**) Accumulation of target proteins after proteasome inhibition. Cells transfected with different constructs were treated with the proteasome inhibitor MG132 for 0, 2, 4, or 6 h. Target protein levels were analyzed by western blotting, with GAPDH used as the internal loading control. Mean values ± SD of three independent biological experiments. (**C**) Ubiquitination levels of subcellularly targeted fusion proteins. Equal amounts of transfected cells were lysed and subjected to immunoprecipitation using anti-FLAG antibodies. Vector and GFP constructs served as negative controls. Mean values ± SD of three independent biological experiments. Numbers at the bottom are the values of ubiquitination levels of Lifeact-LaG or LaG-memb relative to that of LaG, which is set to 1.00.

In the comparative study examining how different subcellular localizations affect the intracellular stability of exogenous proteins, endomembrane and the cytoskeleton localization motifs exhibited the most pronounced accumulation effects. To elucidate the underlying mechanisms of this phenomenon, we first investigated the degradation kinetics of exogenous proteins within the cell. Plasmids encoding endomembrane- or cytoskeleton localization motifs were transiently transfected into 293T cells, followed by treatment with the protein synthesis inhibitor cycloheximide (CHX) for varying time intervals. All four exogenous proteins examined in this study displayed similar degradation profiles. Between 0 and 6 h of CHX treatment, both endomembrane- and cytoskeleton-targeted fusion proteins exhibited slower degradation rates compared to their cytosolic counterparts. Notably, proteins targeted to the endomembrane system were virtually resistant to degradation, demonstrating remarkable stability (Figure 3A).

Since the primary pathway for intracellular degradation of exogenous proteins is the ubiquitin-proteasome system, we further treated cells with the proteasome inhibitor MG132. This resulted in the accumulation of both the cytosolic antibody and the cytoskeleton-targeted antibody, while the endomembrane-targeted antibody exhibited negligible changes, indicating that it was largely unaffected by proteasomal degradation (Figure 3B). Consistently, assessment of the ubiquitination levels of these antibodies revealed that targeting to either the cytoskeleton or the endomembrane substantially reduced the degree of ubiquitination in the cytosol. Among them, the endomembrane-targeted antibody exhibited the lowest ubiquitination level, correlating with its superior stability (Figure 3C).

Collectively, these results demonstrate that redirecting exogenous proteins from the cytosol to either the endomembrane or cytoskeleton significantly enhances their intracellular stability, thereby contributing to their increased accumulation within the cell.

### 2.4. Localization Motifs Do Not Impair Cytoplasmic Function of Exogenous Proteins

**Figure 4:**
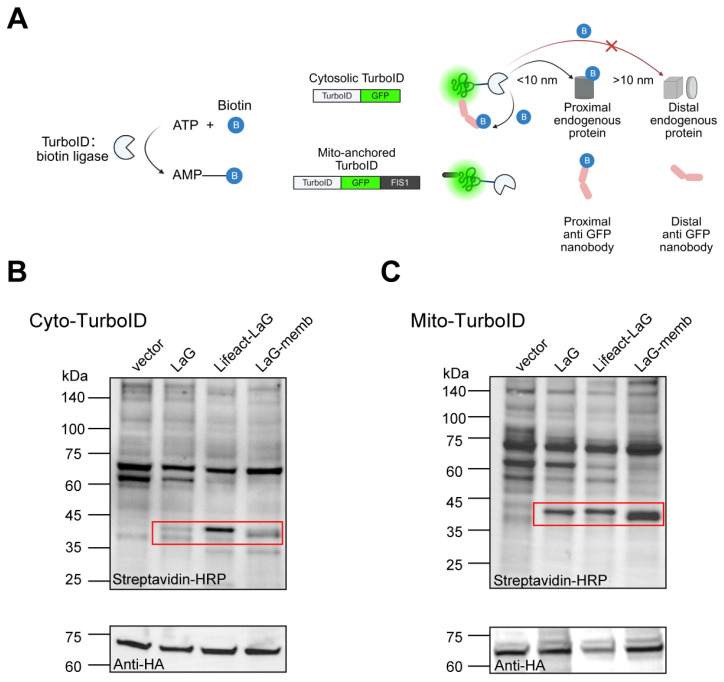
Altered antibody localization does not impair antigen binding. (**A**) Schematic of proximity labeling. TurboID utilizes ATP and biotin to generate biotin–5′-AMP, a reactive intermediate capable of covalently labeling endogenous proteins within a <10 nm radius. We constructed GFP fused to TurboID and targeted it to either the cytosol or mitochondrial surface to assess nearby antibody levels. Created with BioRender (https://www.biorender.com/, accessed on 4 September 2025). (**B**) Antibody proximity levels for cytosolic antigen. HEK-293T cells were co-transfected with antigen and antibody plasmids for 72 h, followed by 10-min treatment with 50 μM biotin. Cell lysates were subjected to WB analysis to detect biotinylated proteins using streptavidin-HRP. HA was used to monitor antigen expression, while the vector group represents endogenous proteins near the antigen in the absence of antibody. Red boxes highlight the molecular weights of antibodies fused to different localization motifs. (**C**) Antibody proximity levels for mitochondria-anchored antigen. HA indicates antigen expression levels, with the vector group showing endogenous proteins near the antigen without antibody presence. Red boxes mark the sizes of antibodies with distinct localization signals.

We have proved that altering the subcellular localization of exogenous proteins in the cytosol can enhance their stability. Most proteins, particularly antibodies, rely on interactions with other proteins to function. However, whether artificially changing a protein’s localization affects its binding to other proteins and thus its functionality remains unclear. Therefore, we employed proximity labeling to investigate how altered localization impacts protein–protein interactions. Compared to traditional methods like co-immunoprecipitation (Co-IP) or yeast two-hybrid (Y2H), proximity labeling (e.g., TurboID) allows us to target a labeling enzyme to specific organelles, generating markers near the protein of interest to capture neighboring interacting molecules. This approach better reflects protein interactions within the cellular microenvironment, making it ideal for our study. Here, we used GFP as an antigen and its dimeric nanobody LaG as a model system to assess how spatial positioning affects antibody accessibility. We localized GFP either freely in the cytosol or anchored to the mitochondrial surface to evaluate how LaG’s subcellular localization influences its function. By fusing TurboID to GFP, we biotinylated nearby proteins, then identified them using streptavidin-HRP to quantify LaG binding (Figure 4A).

Under conditions ensuring equal GFP expression, we compared the levels of correctly biotinylated LaG. We found that endomembrane- or cytoskeleton-localized LaG still bound GFP effectively. In fact, their binding was significantly enhanced compared to free cytosolic LaG. Here we observed distinct interaction preferences depending on the subcellular localization of both the antigen and antibody: cytoskeleton-targeted LaG demonstrated the strongest binding affinity for cytosolic GFP, while endomembrane-localized LaG exhibited more frequent interactions with mitochondria-anchored GFP (Figure 4B,C). These findings suggest that optimal antibody–antigen recognition depends on proper spatial matching between the target localization and antibody positioning within cellular compartments. These results demonstrate that redirecting exogenous proteins from the cytosol to endomembrane or the cytoskeleton not only preserves functionality but also improves stability and cellular accumulation, enhancing their performance.

### 2.5. Localization Motifs Prolong the Function of Exogenous Proteins

**Figure 5:**
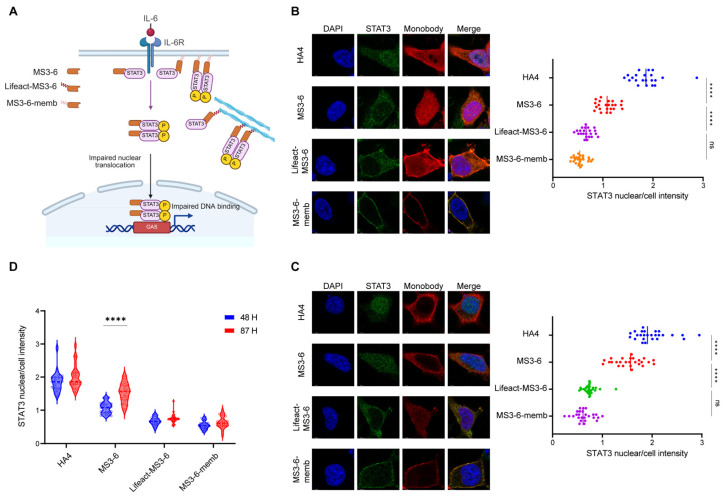
Antibodies fused with cytoskeletal or endomembrane localization motifs exhibit prolonged inhibitory effects. (**A**) Schematic illustration showing that how MS3-6 with different subcellular localizations reduces STAT3 nuclear enrichment upon IL-6 stimulation. Created with BioRender (https://www.biorender.com/, accessed on 4 September 2025). (**B**,**C**) Confocal microscopy images of HEK293 cells expressing MS3-6 at 48 h (**B**) and 87 h (**C**) post-transfection, following IL-6 treatment to assess STAT3 nuclear translocation. HA4 (a non-binding monobody) served as the negative control. Scale bar: 6 µm. Nuclei (blue) and cellular regions (red) were demarcated using Image J. The number of individual cells analyzed (*n*) is over 20. Right panels show quantification of STAT3 nuclear/whole-cell fluorescence ratios. Statistical analyses in (**B**) (**** *p* < 0.0001 and ns = 0.1129) and (**C**) (**** *p* < 0.0001 and ns = 0.3523) were carried out with one-way ANOVA followed by Tukey’s multiple comparisons test. (**D**) Violin plots comparing STAT3 nuclear/cytoplasmic distribution ratios from (**B**,**C**), demonstrating the time-dependent efficacy of MS3-6 in blocking STAT3 nuclear translocation. Blue: 48 h post-transfection; red: 87 h post-transfection. Statistical analyses of MS3-6 proteins (**** *p* < 0.0001) were carried out with two-way ANOVA followed by Tukey’s multiple comparisons test.

Our previous work has established that modifying intracellular antibodies with specific localization motifs significantly improves their stability and increases local concentration at antigen-binding sites. To further investigate whether this elevated antibody level translates to enhanced functional performance, we focused on STAT3 (Signal Transducer and Activator of Transcription 3), a critical mediator of the JAK-STAT signaling pathway involved in numerous physiological and pathological processes. The constitutive activation of STAT3 is known to persistently express downstream genes that drive tumorigenesis, autoimmune diseases, and various metabolic disorders [39,40]. Therefore, blocking the translocation of STAT3 from the cytoplasm to the nucleus holds significant therapeutic importance. The engineered monobody MS3-6 has been previously reported to bind the N-terminal domain of STAT3, effectively preventing its nuclear translocation and subsequent DNA binding, thereby suppressing its transcriptional activation capability. This makes the STAT3-MS3-6 system an ideal model for studying how subcellular targeting affects antibody functionality. Using immunofluorescence microscopy with DAPI nuclear counterstaining, we quantitatively assessed STAT3 nuclear translocation by calculating the nuclear-to-whole cell fluorescence intensity ratio (nuclear/cell). Our results showed that IL-6 stimulation induced robust STAT3 nuclear accumulation (nuclear/cell ratio ~2), while MS3-6 treatment effectively blocked this translocation (reducing the ratio to ~1), consistent with published data. Comparative analysis revealed that MS3-6 antibodies fused with localization peptides demonstrated superior inhibitory performance compared to their free counterparts. The targeted antibodies maintained STAT3 in the cytoplasm more effectively, confirming that the addition of localization peptides not only preserves but enhances antibody–antigen interaction (Figure 5A,B). Importantly, these modifications did not compromise the antibody’s binding affinity for STAT3. Long-term culture experiments (87 h) demonstrated the critical advantage of localization-tagged antibodies. While free MS3-6 showed significant functional decline due to protein degradation, the targeted versions maintained their inhibitory capacity against STAT3 nuclear translocation (Figure 5C,D). This stability advantage was particularly evident for endomembrane- and cytoskeleton-targeted MS3-6 variants, which retained higher intracellular concentrations over extended periods. The successful application of this approach to STAT3 inhibition suggests broad potential for intracellular antibody engineering against other challenging targets. These findings demonstrate that strategic subcellular localization of therapeutic antibodies can simultaneously improve their stability, prolong functional duration, and enhance target engagement.

## 3. Discussion

The intracellular environment presents unique challenges for the stability and functionality of exogenous proteins, including therapeutic antibodies and nanobodies. While nanobodies offer distinct advantages over conventional antibodies due to their small size and exceptional stability, their application in intracellular settings has been limited by rapid degradation in the reducing environment of the cytosol. Previous strategies to enhance intracellular stability have focused on optimizing protein charge or fusion to solubilizing tags [15,17], yet these approaches often fail to address the fundamental issue of proteasomal targeting.

Actin, a well-known intracellular structural protein, has been reported to exhibit a long half-life and high stability [26]. We found that linking exogenous proteins to actin through protein–protein interaction enhances their intracellular stability. This strategy reduces the level of ubiquitination of the exogenous protein, as confirmed by our experiments. Interestingly, we observed that adding localization motifs directing the protein to the endomembrane system resulted in even lower ubiquitination levels than actin targeting. HRAS, a member of the small GTPase family, is strongly associated with the plasma membrane and endomembrane compartments due to post-translational modifications (PTMs) on its C-terminal CAAX motif, including prenylation and palmitoylation [34,41,42]. These lipid modifications also play roles in regulating protein stability. For example, farnesylation has been shown to significantly increase the stability of the Ykt6 protein in extracellular settings [43]. Similarly, palmitoylation protects proteins such as PD-L1 [44], Oct4 [45], CLDN3 [46], and Fas [47] from lysosomal degradation [48]. However, in some cases, these same modifications are linked to reduced protein half-life [49,50,51,52]. Therefore, more studies are needed to clarify the role of lipid PTMs in controlling the stability of exogenous proteins. In addition, anchoring proteins to the endomembrane system or the cytoskeleton can potentially impact cellular activities. For example, ER stress can activate cell death mechanisms such as apoptosis or autophagy. The cytoskeleton is essential for maintaining cell shape, supporting cell migration, and facilitating division. The selection of specific proteins and their anchoring configurations to the cell endomembrane system or cytoskeleton requires rigorous design and validation.

The rapid advancement of mRNA-based therapies in clinical settings offers a safe and translatable strategy for delivering nanobodies into cells. One major challenge in mRNA therapy is the transient nature of protein expression, which limits therapeutic duration. To overcome this, extensive research has focused on chemically modifying mRNA to extend its intracellular half-life and prolong dosing intervals. Our study significantly expands the toolkit for intracellular antibody engineering, with immediate implications for therapeutic nanobody design and fundamental studies of protein homeostasis. The ability to prolong nanobody half-life at the post-translational level while maintaining function opens new possibilities for targeting intracellular proteins involved in cancer, neurodegeneration, and other diseases where sustained target engagement is critical.

## 4. Materials and Methods

### 4.1. Plasmid Construction

Briefly, to investigate the effect of subcellular localization on protein stability, localization motifs directing proteins to different subcellular compartments were fused to either the N- or C-terminus of the protein of interest (d1GFP, LaG, Nb and MS3-6 protein). The C-terminal sequences derived from Fis1 and HRAS proteins were added to the C-terminus of the target protein to direct localization to the mitochondrial outer membrane or intracellular membrane system, respectively. For endoplasmic reticulum (ER) surface localization, the ER-targeting motif of cytochrome P450 was fused to the N-terminus of the target protein, ensuring its ER surface orientation facing the cytosol. Another N-terminal fusion was Lifeact, a short peptide that binds filamentous actin (F-actin), facilitating cytoskeletal targeting. To enable intracellular detection, a 3 × FLAG tag was incorporated at the N-terminus of the target protein. For flow cytometry analysis, the mCherry reporter was separated from the target protein by an IRES sequence. In proximity labeling experiments, the TurboID biotin ligase sequence was fused to the N-terminus of GFP, while its subcellular localization was altered by adding the C-terminal transmembrane domain of Fis1 at the GFP C-terminus. All synthetic DNA fragments and primers were purchased from Azenta Life Sciences (Suzhou, China) and subcloned into the pcDNA3.1 backbone, with transgene expression driven by the CMV promoter. PCR amplification reagents (cat#P510) were obtained from Vazyme Biotech Co., Ltd. (Nanjing, China). DNA ligation kits (cat#NR005) were sourced from Novoprotein Scientific Inc. (Suzhou, China). Top10 competent cells (cat#DL1010) for transformation were purchased from WeidiBio (Shanghai, China). All plasmids were sequencing-verified and are available upon request.

### 4.2. Cell Culture and Transfection

Human embryonic kidney HEK293T cells were cultured in Dulbecco’s Modified Eagle Medium (DMEM) (cat# MA0212, MeilunBio, Dalian, China) supplemented with 10% fetal bovine serum (FBS) (cat# 40131ES76, Yeasen, Shanghai, China) and 1% penicillin/streptomycin (PS) (cat# MA0110, MeilunBio, Dalian, China). Cells were maintained in a humidified incubator at 37 °C with 5% CO_2_.

### 4.3. Transfection Procedure

One day prior to transfection, cells were seeded in 6-well plates. For each well, 200 μL of a transfection mixture containing 5 μg plasmid DNA and 2 μL Vigofect transfection reagent (cat# T001, Vigorous Biotech, Beijing, China) was added dropwise. After 4–6 h, the medium was replaced with fresh complete DMEM.

### 4.4. MG132 Treatment

At 24 h post-transfection, the medium was replaced with complete DMEM containing 10 μM MG132 (cat# HY-13259, MedChemExpress, Monmouth Junction, NJ, USA). Cells were harvested at specified time points, lysed on ice using RIPA buffer (cat# P0013B, Beyotime, Shanghai, China), and centrifuged at 13,000× *g* for 30 min at 4 °C. The supernatant was collected for Western blot analysis.

### 4.5. CHX (Cycloheximide) Treatment

At 24 h post-transfection, cells were first treated with 10 μM MG132 in complete DMEM for 4 h to block protein degradation. The medium was then replaced with DMEM containing 50 μg/mL CHX (cat# HY-12320, MedChemExpress, Monmouth Junction, NJ, USA) and 1% PS. Cells were collected at designated time points for flow cytometry analysis.

### 4.6. Flow Cytometry Analysis

Following transfection, harvested cells were fixed in fixation buffer (cat#420801, Biolegend, San Diego, CA, USA) at 37 °C for 20 min in the dark, then washed twice with Intracellular Staining Permeabilization Wash Buffer (cat#421002, Biolegend, San Diego, CA, USA). Cells were stained with APC-conjugated anti-FLAG antibody (cat#637308, Biolegend, San Diego, CA, USA) for 20 min at room temperature. Analysis was performed using a BD LSR Fortessa flow cytometer, where the mean fluorescence intensity (MFI) in the APC channel of mCherry-positive cells represented the intracellular target protein level, enabling assessment of protein accumulation and degradation kinetics.

### 4.7. Western Blot Analysis

The treated cell samples were digested with trypsin (cat#MA0233, MeilunBio, Dalian, China) and centrifuged at 1500 rpm for 5 min to pellet the cells. After supernatant removal, the pellets were lysed with RIPA buffer on ice for 30 min, followed by centrifugation at 13,000× *g* for 10 min at 4 °C to clarify the lysates. The clarified supernatants were transferred to new microcentrifuge tubes.

Protein concentration was determined using a BCA protein assay kit (cat#23227, Thermo Fisher Scientific, Waltham, MA, USA). An appropriate amount of supernatant was mixed with 5× SDS-PAGE loading buffer (cat#P0015, Beyotime, Shanghai, China) and boiled at 100 °C for 10 min. Approximately 20 μg of protein per sample was separated on 4–20% SDS-PAGE gels (cat#SLE015, Smart-Lifesciences, Changzhou, China) and transferred onto polyvinylidene fluoride (PVDF) membranes (cat#ISEQ00010, Merck, Billerica, MA, USA).

The PVDF membranes were blocked with 5% skim milk in TBST at room temperature for 1 h, followed by overnight incubation at 4 °C with primary antibodies diluted in 5% skim milk: anti-FLAG (cat#F1804, Sigma, St. Louis, MI, USA, 1:2000), anti-Ubiquitin (cat#3936T, CST, Danvers, MA, USA, 1:1000), anti-HA tag (cat#66006-2-Ig, Proteintech, Wuhan, China, 1:5000), and anti-GAPDH (cat#60004-1-Ig, Proteintech, Wuhan, China, 1:5000).

After three 5-min TBST washes, the membranes were incubated for 1 h at room temperature with secondary antibodies diluted in 5% skim milk: goat anti-mouse (cat#SA00001-1, Proteintech, Wuhan, China, 1:5000) or goat anti-rabbit (cat#SA00001-2, Proteintech, Wuhan, China, 1:5000). Following three additional 5-min TBST washes, the membranes were developed using ECL reagent (cat#P0018AFT, Beyotime, Shanghai, China).

### 4.8. Ubiquitination Assay

HEK293T cells transiently transfected with target plasmids for 40 h were treated with 10 μM MG132 for 4 h. The treatment was terminated by rapid washing with ice-cold PBS. After discarding the medium, adherent cells were scraped in ice-cold PBS and transferred to pre-chilled centrifuge tubes, followed by centrifugation at 1500 rpm for 5 min at 4 °C. The cell pellets were washed twice with ice-cold 1× PBS. Total proteins were extracted using RIPA lysis buffer. Meanwhile, Anti-Flag Magarose Beads (cat#KTSM1338, AlpalifeBio, Shenzhen, China) were pre-equilibrated with three PBST washes. The clarified protein lysates were then incubated with the equilibrated beads at 4 °C for 3 h with rotation. After magnetic separation until the supernatant became clear, the supernatant was discarded. The beads were thoroughly washed at least four times with PBST to remove nonspecific binding. Finally, the beads were resuspended in SDS loading buffer and boiled at 95 °C for 10 min. The eluted proteins in the supernatant were collected by magnetic separation and subjected to subsequent western blot analysis.

### 4.9. Proximity Labeling Assay

Following 72-h transient transfection of target plasmids into HEK293T cells, biotin (cat# HY-B0511, MedChemExpress, Monmouth Junction, NJ, USA) was added at a final concentration of 50 μM for 1-min pulse labeling. The labeling reaction was immediately quenched by five rapid washes with ice-cold PBS. The harvested cell pellets were then lysed for subsequent processing using standard western blot procedures. For detection of biotinylated proteins, streptavidin-HRP (cat#434323, Invitrogen, Carlsbad, CA, USA) was diluted in 3% BSA and incubated with PVDF membranes at room temperature for 30 min.

### 4.10. Immunofluorescence Staining

One day prior to transfection, 293T cells were seeded on coverslips (cat#BS-09-RC, Biosharp, Shanghai, China). For colocalization experiments between localization motifs and subcellular markers, cells were processed 24 h post-transfection. For STAT3 nuclear translocation inhibition assays with MS3-6, transfected cells were stimulated with IL-6 (100 ng/mL) at 37 °C for 20 min before processing. Then the cells were washed twice with PBS and fixed with either ice-cold methanol for 10 min at room temperature, or 4% paraformaldehyde (PFA) for 15 min and followed by three PBS washes. Following permeabilization with 0.1% Triton X-100 in PBS for 5 min at room temperature and three PBS washes, cells were blocked with 5% BSA in PBS for 1 h at room temperature before overnight incubation at 4 °C with primary antibodies diluted in 5% BSA/PBS: anti-Calreticulin (ER marker, 1:400; #ab92516, Abcam, Cambridge, MA, USA), anti-COX IV (mitochondrial outer membrane, 1:200; #4850T, CST, Danvers, MA, USA), anti-Ras (membrane system, 1:200; CST #91054S), anti-FLAG (1:400; Sigma #F1804), and anti-STAT3 (1:800; CST #12640s). After three PBS washes, cells were incubated for 1 h at room temperature with secondary antibodies: CoraLite488-conjugated goat anti-rabbit IgG (1:200; #SA00013-2, Proteintech, Wuhan, China), CoraLite Plus 594-conjugated goat anti-mouse IgG (1:200; #RGAM004, Proteintech, Wuhan, China), and CoraLite488-conjugated goat anti-mouse IgG (1:200; #SA00013-1, Proteintech, Wuhan, China), followed by nuclear staining with DAPI (1:1000; #C1002, Beyotime Shanghai, China) and F-actin staining with fluorescein phalloidin (1:1000; #HY-K0902, MedChemExpress, Monmouth Junction, NJ, USA). After five PBS washes, samples were imaged using a Leica STELLARIS 5 confocal microscope, Wetzlar, Germany. Analysis was performed with LAS X 4.5.0 software: Colocalization: Pearson’s coefficient calculated using the colocalization module to analyze STAT3 nuclear translocation. The nuclei were defined by Hoechst staining. Cell boundaries were determined by anti-mouse CoraLite Plus 594 signal STAT3 (anti-rabbit CoraLite488) intensity quantified in nuclear vs. whole-cell regions. Data are expressed as nuclear/total STAT3 ratio. Each sample contains ~30 cells per plasmid group from two independent experiments.

### 4.11. Statistical Analysis

All statistical analyses were performed using GraphPad Prism 9 software. The sample size (*n*) and data representation methods are specified in the corresponding figure legends. Statistical significance is denoted as follows: **** *p* < 0.0001, *** *p* < 0.001, ** *p* < 0.01, * *p* < 0.05, and “ns” indicates non-significant differences.

## 5. Conclusions

By anchoring nanobodies to the endomembrane system or cytoskeleton, we achieved a 2- to 3-fold increase in intracellular accumulation, primarily by reducing ubiquitination-mediated degradation. Notably, this strategy not only preserved but also enhanced antigen-binding efficacy, as validated by proximity labeling and functional assays targeting STAT3 nuclear translocation. These findings establish a generalizable approach to overcome the intrinsic instability of cytoplasmic nanobodies, offering a protein-level solution that could complement existing mRNA-based delivery strategies.

## Data Availability

The data supporting the findings of this study are available from the corresponding author upon reasonable request.
